# Joining the gender war online: entitativity, negative emotion, and engagement with gender-polarized posts

**DOI:** 10.3389/fpsyg.2026.1961949

**Published:** 2026-10-26

**Authors:** Yaxin Li, Zhou Fang, Yong Bian

**Affiliations:** 1 Claremont Graduate University, Claremont, CA, United States; 2 University of Charleston, Charleston, WV, United States; 3 Wayne State University, Detroit, MI, United States; 4 Zhejiang Gongshang University, Hangzhou, China

**Keywords:** attitude generalization, entitativity, gender conflict, intergroup perception, rednote, social media, zhihu

## Abstract

**Introduction:**

Gender-polarized discourse has become increasingly visible on social media, where interpersonal conflicts are often generalized to broader gender groups. This study examined the associations of perceived group entitativity and negative intergroup emotion with individuals' responses to gender-polarized framing in online discourse.

**Methods:**

A total of 800 Chinese adults were exposed to gender-conflict posts presented in RedNote-like or Zhihu-like social-media contexts. The posts described interpersonal conflicts and explicitly generalized these incidents to the target gender group. Participants subsequently reported their perceived group entitativity, negative intergroup emotion, and responses to gender-polarized framing.

**Results:**

Perceived group entitativity was strongly and positively associated with responses to gender-polarized framing, indicating greater alignment with negative group-based narratives among participants who perceived the target gender as a coherent social entity. Negative intergroup emotion was also positively associated with these responses, although the hypothesized indirect effect of entitativity through negative emotion was not supported. Responses differed modestly between the two gender-pairing conditions, whereas neither the main effect of platform context nor the gender-pairing × platform interaction was significant.

**Discussion:**

These findings suggest that perceived group coherence and negative intergroup emotion are associated with individuals' alignment with gender-polarized narratives on social media. The results highlight the importance of distinguishing the initial emergence of group generalization from psychological responses to generalizations already embedded in online discourse.

## 1 Introduction

Gender-related conflict has become increasingly visible on Chinese social media, where discussions of interpersonal experiences can develop into broader negative evaluations of men or women as social groups (Hu et al., 2021; Peng et al., 2021). In the present study, we refer to posts containing this form of individual-to-group generalization as gender-polarized posts. Specifically, gender-polarized posts are social-media posts that frame a negative interpersonal experience involving a particular man or woman as indicative of men or women as a broader social category. The term therefore refers not simply to controversial or gender-related content, but to a discursive shift from criticism of a specific individual to a broader negative evaluation of the gender group.China therefore provides a particularly relevant context for examining online gender conflict, as highly visible gender-related discussions frequently extend beyond individual interpersonal experiences into broader evaluations of men or women as social groups. Research on Chinese digital discourse has documented increasingly polarized representations of gender and feminism, including the use of derogatory labels and adversarial framings (Bao, 2023; Qin et al., 2024; Yiwei, 2024).

The Chinese context is particularly informative because contemporary online gender conflict in China is situated within both a broader East Asian trajectory of changing gender relations and an increasingly transnational digital environment (Sun, 2026). Some of the tensions now visible in Chinese online discourse—including disputes surrounding dating and marriage, expectations of men and women, household responsibilities, and antagonistic representations of the opposite gender—resemble patterns previously documented in Japan and, particularly, South Korea, where rapid socioeconomic and demographic change has accompanied highly visible gender conflict and anti-feminist or feminist backlash (Chen, 2025; Zhao et al., 2026). China should not be assumed to follow the same trajectory, but these parallels suggest that contemporary Chinese gender polarization can be understood as part of a broader process through which changing gender roles and persistent social expectations become translated into group-based conflict.

At the same time, the relevance of the Chinese case increasingly extends beyond China itself. Chinese-origin digital platforms and platform cultures have become increasingly visible internationally. TikTok, the international counterpart of Douyin, has become a major component of the global social-media environment, while RedNote (Xiaohongshu) attracted a sudden influx of overseas users during the 2025 TikTok refugee phenomenon (Badurak and Ramirez, 2026). The resulting cross-cultural encounters—including the widely discussed China-U.S. account reconciliation (中美对账), in which Chinese and American users compared everyday experiences, costs, work, family life, and social expectations—illustrate how platform-specific discourses that were once largely embedded within Chinese digital culture can rapidly enter transnational communication (Hongchen et al., 2025). Consequently, understanding how users respond to gender-polarized narratives on Chinese platforms is relevant not only to Chinese social psychology but also to the increasingly global circulation and interpretation of such narratives.

This perspective may also be informative for societies in which comparable transformations in gender roles and digital discourse are at an earlier or different stage. The parallels among Japan, South Korea, and China suggest that interpersonal tensions associated with changing expectations of relationships, marriage, work, and family can, under some conditions, become reframed as conflicts between gender groups (Jeong and Lee, 2018; MARTIN, 1990). Rather than treating China as either culturally unique or as a universal model, the present study therefore uses the Chinese case to examine a potentially transferable psychological process: how an interpersonal incident becomes meaningful as information about an entire gender category, and why some individuals are more receptive than others to such group-based framing. Comparative research across East Asian, Western, and other developing or rapidly changing societies will be necessary to determine which aspects of this process are culturally specific and which are more general.

In Chinese internet slang, the term daquan (打拳, throwing punches) is sometimes used to describe aggressive online participation in gender conflict, particularly the posting of provocative or extreme statements that attack or disparage men or women as a group (Chen and Zheng, 2023). Although colloquial and often derogatory, the metaphor of throwing punches vividly captures the adversarial nature of such discourse, in which users may not merely observe gender conflict but actively align with and reinforce it. Such discourse illustrates an important social-psychological phenomenon: individuals encountering gender-polarized posts may differ in the extent to which they align with and reinforce the broader group-based framing presented in those posts. Understanding this process is important because engagement with gender-polarized discourse can contribute to stereotyping, intergroup hostility, and polarization in online environments.

Gender polarization is part of a broader transformation in contemporary gender relations that has attracted increasing attention across social and cultural contexts. Recent research has documented both feminist mobilization and countervailing forms of gender backlash, including resistance to changing gender norms, anti-feminist attitudes, misogynistic discourse, and increasingly adversarial understandings of relations between men and women (Napier and Kusano, 2026). These tensions are visible offline in attitudes toward gender roles, employment, marriage, and family life, but digital environments provide particularly favorable conditions for their expression and circulation (Jang and Nanditha, 2025; Neske, 2025). Social media can transform personal experiences into collectively interpreted narratives, allowing individual incidents involving dating, relationships, work, or family to be presented as evidence of broader problems with “men” or “women” (Gerbaudo, 2024).

Importantly, much of this literature has focused on the content, production, and circulation of polarized gender discourse or on the social and political conditions associated with gender backlash (Aliyah et al., 2026). Comparatively less attention has been paid to the social-cognitive processes through which audiences respond to such discourse once it is encountered. In particular, why does the same gender-generalizing narrative appear compelling to some individuals but not to others? The present study addresses this question by focusing on perceived group entitativity and negative intergroup emotion as two psychological factors potentially associated with receptivity to gender-polarized framing.

One social-cognitive mechanism that may facilitate engagement with gender-polarized discourse is perceived group entitativity, or the extent to which a collection of individuals is perceived as a coherent and unified social entity (Campbell, 1958; Lickel et al., 2000). When a group is perceived as highly entitative, its members are more likely to be viewed as similar, interconnected, and representative of the group as a whole. Consequently, information about an individual member may be treated as more diagnostic of the broader group, increasing the likelihood that evaluations of that individual are generalized to other group members (Crawford et al., 2002; Newheiser and Dovidio, 2012; Yzerbyt et al., 1998). Recent experimental evidence further demonstrates that generalization from an individual outgroup member to the broader outgroup is stronger when the outgroup is perceived as more entitative (Neji et al., 2025). This mechanism may be particularly relevant to gender categories because gender constitutes a highly salient social category and frequently provides a basis for social categorization and stereotype-based inference. This tendency is not limited to intergroup contact. Recent research on collective blame shows that perceptions of a social group as highly entitative can facilitate the extension of responsibility for an individual member's behavior to the broader group (Alam et al., 2025). In online gender conflict, therefore, perceptions of men or women as coherent social groups may influence the extent to which individuals align with and reinforce gender-polarized interpretations of interpersonal conflict.

An important unresolved question concerns the role of emotion in this process. Intergroup research has demonstrated that negative group-directed emotions, including anger, fear, contempt, and distrust, can shape evaluations and behavioral intentions toward social groups (Mackie et al., 2000; Paolini et al., 2004). From an affective account, perceiving a group in particular ways may generate negative emotional responses, which subsequently increase individuals' engagement with gender-polarized discourse. However, a cognitive account suggests that perceived group structure may facilitate such engagement more directly: when individuals perceive members of a category as constituting a coherent entity, they may be more receptive to posts that frame individual conflicts in broader group-based terms even without emotion serving as the intervening mechanism. Distinguishing these possibilities is important for understanding whether engagement with online gender conflict primarily reflects affective reactions to conflict or a more fundamental process of group-based social inference.

These processes may also depend on the social-media environment in which information is encountered. Digital platforms differ in their user populations, communication norms, interfaces, and dominant forms of interaction, all of which may shape how social information is interpreted. The present study focuses on Zhihu and RedNote (Xiaohongshu), two prominent Chinese social-media platforms with distinct communicative environments. Zhihu is primarily a text-based knowledge-sharing platform characterized by extended discussion and argumentation, whereas RedNote is a visually oriented lifestyle platform in which personal narratives and community interaction play a prominent role (Chen et al., 2018; Gan, 2024). Recent evidence from Chinese social media further suggests that gender antagonism is not uniform across digital environments. A large-scale cross-platform analysis of more than 100,000 posts and comments found that gendered antagonism takes distinct forms across Weibo, Zhihu, and Xiaohongshu, reflecting differences in platform affordances, community norms, and governance structures (Su et al., 2026). Comparing these contexts provides an opportunity to examine whether platform context influences individual's engagement with gender-polarized discourse.

Recent evidence nevertheless suggests that the role of entitativity may depend on the type of group judgment involved. Whereas, perceived outgroup entitativity can facilitate generalization from individual members to the broader group (Neji et al., 2025), experimentally increasing ingroup entitativity did not consistently alter judgments of deviant group members or their culture in other contexts (Khosrowtaj et al., 2024). These findings highlight the need to examine whether entitativity similarly shapes responses to gender-polarized discourse when the target is a highly salient social category such as gender.

Accordingly, the present study examines engagement with gender-polarized posts that frame interpersonal conflicts in broader gender-group terms. Rather than asking how such group-level generalization initially emerges, the study focuses on why individuals differ in their willingness to align with and reinforce an already polarized framing of gender conflict. We investigate whether such responses are associated with perceived entitativity and negative intergroup emotion and whether they differ across platform and gender-pairing contexts.We propose the following hypotheses:

**H1:** Higher perceived entitativity will be associated with stronger engagement with gender-polarized posts.

**H2:** Negative intergroup emotion will mediate the relationship between perceived entitativity and engagement with gender-polarized posts.

**H3**: Engagement with gender-polarized posts will differ across platform and gender-pairing conditions, with stronger engagement expected in the RedNote condition than in the Zhihu condition and in the female-target/male-participant condition than in the male-target/female-participant condition.

The remainder of this paper is organized as follows. Section 2 reviews relevant theoretical frameworks concerning entitativity, intergroup emotion, and engagement with gender-polarized discourse, particularly in digital contexts. Section 3 outlines the experimental design, materials, and analytic strategy. Section 4 presents the main results, including ANOVA and mediation analyses. Section 5 discusses the theoretical and practical implications of the findings, and Section 6 concludes with suggestions for future research.

## 2 Background

### 2.1 Group entitativity

The concept of group entitativity was originally proposed by Campbell (Campbell, 1958) to describe the degree to which a set of individuals is perceived as a coherent and unified entity. Later empirical work (Lickel et al., 2000) operationalized entitativity as a function of shared goals, similarity, interaction, and the perceived permanence of a group. High-entitativity groups, such as families or sports teams, are more likely to be perceived as acting with a collective will; in contrast, low-entitativity groups (e.g., people in a cafeteria) are seen as aggregates.

Entitativity plays a crucial role in shaping social judgments. When a group is perceived as high in entitativity, observers are more likely to generalize from an individual's behavior to the group as a whole (Newheiser and Dovidio, 2012; Yzerbyt et al., 1998). Recent research further clarifies that entitativity should be distinguished from its antecedents (e.g., similarity, interaction, shared goals) and from its outcomes (e.g., stereotyping, generalization). Blanchard et al. (2020) developed a validated measure that demonstrates this distinction across both online and face-to-face groups. In addition, entitativity has been shown to increase under conditions of uncertainty, thereby strengthening group identification and stereotyping tendencies (Hogg et al., 2007). These findings suggest that entitativity may also shape how individuals respond when online content explicitly frames interpersonal conflict in group-based terms.

This process facilitates stereotype formation and moral judgment, especially in intergroup contexts. In the domain of gender conflict, perceived group unity make individuals more receptive to posts that portray interpersonal conflicts as representative of broader gender-group characteristics (Crawford et al., 2002). As gender is already a socially salient and chronically accessible category (Fiske et al., 1991), gender-polarized content may be particularly susceptible to entitativity-based group judgments in emotionally charged digital contexts.

### 2.2 Gender polarization and gender backlash

Gender polarization has become an increasingly visible feature of contemporary gender relations across different social and cultural contexts. Recent cross-national evidence indicates that men and women can diverge substantially in their attitudes toward gender-related issues, although the magnitude and direction of such differences vary across societies (Napier and Kusano, 2026). These divisions have emerged alongside broader changes in gender roles and continuing debates over feminism and gender equality. Resistance to such changes is often discussed in terms of gender backlash, which may involve resistance to changing gender norms, anti-feminist attitudes, misogynistic discourse, and opposition to feminist mobilization (Faludi, 2009; MacDonald et al., 2026; Roberts and Wescott, 2026; Walby, 2003). In some contexts, these conflicts have developed into broader “gender culture wars,” in which disagreements over gender extend beyond particular policies or behaviors and become organized around competing social identities and understandings of gender relations (Graff and Korolczuk, 2021; Gur-Ze'ev and Kligler-Vilenchik, 2026). In China, related tensions are evident in increasingly hostile representations of feminism in online discourse (Bao, 2023, 2024), as well as in associations among modern sexism, misogyny, and attitudes toward feminist engagement (Chen and Zheng, 2023). Comparable tensions have also been documented elsewhere in East Asia, particularly in South Korea, where feminist mobilization has developed alongside highly visible oppositional gender discourse (Jang and Nanditha, 2025; Jeong and Lee, 2018).

Importantly, gender polarization is not confined to digital environments. Differences and conflicts surrounding gender roles, employment, intimate relationships, marriage, family responsibilities, and gender equality are embedded in broader social relations and can be expressed both online and offline (Napier and Kusano, 2026; Nennstiel and Hudde, 2025; Oklobdzija and Witko, 2026). Social media, however, provides distinctive conditions through which such tensions can become highly visible, collectively interpreted, and circulated. Personal experiences involving relationships, work, family, or perceived unfairness can be transformed into collective narratives when individuals connect their experiences to those of others (Cui, 2026; Gerbaudo, 2024; Shan, 2026). Digital environments also expose users to the judgments and emotional reactions of others, which may shape perceptions of the intensity and prevalence of intergroup conflict. For example, social-media users have been shown to overperceive moral outrage expressed by others, thereby inflating perceptions of intergroup hostility(Brady et al., 2023; Jo et al., 2025; Sevincer and Stoet, 2025). The form taken by gender antagonism may also depend on the digital environment in which it occurs. Recent evidence from Chinese social media suggests that gender antagonism differs across platforms as a function of platform affordances, community norms, and governance structures (Su et al., 2026). Online gender polarization can therefore be understood not as a phenomenon separate from offline gender relations, but as one arena in which broader gender tensions are expressed, interpreted, and potentially reinforced.

Existing research on gender polarization and backlash has provided important insights into the social conditions, ideological content, production, and circulation of adversarial gender discourse (Bao, 2023, 2024; Fontanella et al., 2024; Jang and Nanditha, 2025). Comparatively less attention, however, has been directed toward the social-cognitive processes through which audiences respond to gender-polarized content once they encounter it. This distinction is important because the persistence of polarized gender narratives depends not only on those who produce or disseminate them, but also on whether audiences find their group-based framing psychologically compelling. In particular, when an interpersonal incident involving one man or woman is explicitly presented as evidence about men or women more generally, individuals may differ substantially in their willingness to accept and reinforce this individual-to-group interpretation. The present study focuses on this audience-level process by examining whether perceived gender-group entitativity and negative intergroup emotion are associated with individuals' responses to gender-polarized framing.

### 2.3 Engagement with gender-polarized discourse

Attitude generalization refers to the process by which an observer extends evaluations or emotions elicited by one group member to the group as a whole (Wright et al., 1997). Generalization may serve adaptive functions, such as reducing cognitive load or facilitating rapid social decision-making. However, in many cases—particularly those involving negative behavior—it can lead to distorted social judgments, prejudice, and intergroup hostility (Krueger and Didonato, 2008). Although the present study does not examine the spontaneous emergence of such generalization, this literature provides an important basis for understanding why individuals may be receptive to online posts that already frame an interpersonal incident as representative of a broader gender group.

Prior research suggests that generalization is more likely when the target group is perceived as internally consistent, morally relevant (Tetlock et al., 2000), or socially salient. These processes are particularly relevant in online environments, where interpersonal incidents may themselves be presented through explicitly group-based or adversarial frames. When a post portrays the behavior of an individual as indicative of “men” or “women” more broadly, users encounter not only information about the incident but also a ready-made interpretation of that incident in gender-group terms (Hsueh et al., 2015; Suler, 2004; Tian and Menchik, 2016). Individuals may nevertheless differ substantially in the extent to which they accept, endorse, or reinforce such a framing.

While attitude generalization has traditionally been studied in offline contexts (e.g., intergroup contact, media priming), recent work has shown that it is highly relevant in digital communication, particularly where anonymity and algorithmic amplification can distort the perception of representativeness (Schumann et al., 2017). In the present context, the central question is therefore not whether a group-level inference emerges spontaneously, but why some individuals are more willing than others to align with and reinforce a gender-generalizing interpretation that is already present in the online content.

### 2.4 Intergroup emotion

Intergroup emotions are affective responses directed not at individuals, but at social groups (Mackie et al., 2000). These emotions differ from personal emotions in that they are contingent upon social identification and group-based appraisal. For example, one may feel anger not at a specific person, but at “men in general” or “feminists,” depending on the nature of the triggering event and perceived group boundaries.

Theoretically, intergroup emotion has been proposed as a mediator between group cognition and behavioral intention (Paolini et al., 2004). That is, when individuals perceive a group as threatening, unfair, or hostile, they may experience emotions such as fear, contempt, or anger, which in turn shape their behavioral or attitudinal responses (Kuppens and Yzerbyt, 2012), including their willingness to align with or reinforce antagonistic group-based discourse. However, some studies have suggested that cognitive antecedents such as entitativity may exert a more direct influence on such responses, bypassing emotion in situations where the evaluative component is strong or where emotional responses are muted by context (Crawford et al., 2002).

Emotion may be particularly consequential in digital environments, where users can misperceive the intensity of others' affective reactions. For example, Brady et al. (2023) found that social media users systematically overperceived moral outrage expressed by others, which in turn inflated perceptions of intergroup hostility.

In text-based digital environments, especially those involving screenshot-based posts or narrative-based conflict, emotional arousal may be attenuated compared to face-to-face confrontation (Shin et al., 2017). In such settings, observers may rely more heavily on group-based inferences than on visceral emotional reactions, suggesting that entitativity and negative intergroup emotion may represent partially distinct cognitive and affective pathways to engagement with gender-polarized discourse.

### 2.5 Platform and sociotechnical context

Social psychological processes do not occur in a vacuum; they are embedded in sociotechnical systems that structure behavior and perception (Pasmore et al., 2019). Digital platforms vary in interface design, content algorithms, user base, and cultural norms. Even when the substantive content is similar, platform-specific visual and contextual cues may therefore influence how users interpret online conflict.

In the present study, we focus on two platforms that represent distinct digital cultures in China: Zhihu, a text-heavy, knowledge-sharing community known for its analytical tone and frequent ideological confrontation; and RedNote, a visually driven, lifestyle-oriented platform centered around lifestyle, beauty, and emotional support (Bao, 2024; Wang and Chang, 2025). While both platforms host gender-related discourse, they differ in tone, audience, and the visibility of dissenting opinions.

These differences are likely to influence psychological processes such as entitativity perception, emotional arousal, and judgment generalization. For example, on RedNote, posts may be interpreted within an in-group framework, emphasizing empathy and collective experience; on Zhihu, the same content may elicit rational critique or intergroup confrontation. Thus, platform serves not only as a delivery mechanism, but also as a contextual moderator of cognitive and affective processing.

### 2.6 Social identity and SIDE theories

The current study can also be informed by broader frameworks on social identity and deindividuation. Social identity theory (Hogg and Reid, 2006; Postmes et al., 2005; Spears and Postmes, 2015) emphasizes that individuals often define themselves in terms of group memberships, which leads to the assimilation of personal judgments into group-based stereotypes. When gender is salient, interpersonal conflicts may be more readily interpreted through the lens of gender-group membership, potentially increasing receptivity to discourse that frames such conflicts in broader “men vs. women” terms.

Similarly, the Social Identity model of Deindividuation Effects (Postmes et al., 2000; Spears et al., 2002) highlights how anonymity and reduced individuating cues in computer-mediated environments enhance the salience of group identity. SIDE research suggests that in online contexts, observers are more likely to attribute an individual's actions to their group identity rather than to personal characteristics. This perspective dovetails with entitativity theory, as both predict that structural or contextual cues which increase perceptions of “groupness” will increase the prominence of group-based interpretations, potentially making individuals more receptive to gender-polarized framings of interpersonal conflict.

## 3 Method

### 3.1 Participants

A total of 800 adult participants (50% female; age *M* = 24.6, SD = 4.7) were recruited through Wenjuanxing, a widely used online data collection platform in mainland China that provides functions similar to Qualtrics or SurveyMonkey, including randomization, skip logic, and data export. Participant gender was self-reported as male, female, or other. Responses from participants who selected “other” were excluded because the study used a balanced binary-gender sampling design, and recruitment continued until the final sample included 400 male and 400 female participants. Participants were required to be native Chinese speakers and regular users of either Zhihu or RedNote. Sampling quotas were used to ensure approximately equal representation across four experimental conditions: (1) male-target/female-participant on Zhihu, (2) female-target/male-participant on Zhihu, (3) male-target/female-participant on RedNote, and (4) female-target/male-participant on RedNote.

All participants provided informed consent electronically before beginning the study. The study protocol was reviewed and approved by the ethics committee of the lead author's institution.

### 3.2 Design and procedure

The study employed a 2 (Gender Pairing: male-target/female-participant vs. female-target/male-participant)) × 2 (Platform Context: Zhihu vs. RedNote) between-subjects design. Participants were randomly presented with a “social-media screenshot” corresponding to their assigned gender-pairing and platform condition, each representing a real-world scenario involving interpersonal gender conflict. Each scenario was developed in gender-mirrored versions that retained the same underlying interpersonal-conflict theme while using gender-appropriate wording and contextual details. Participant gender and target gender were paired by design: male participants viewed female-target vignettes, whereas female participants viewed male-target vignettes. Thus, participant gender and target gender were not independently manipulated and their effects cannot be statistically disentangled in the present design.

After reading the vignette, participants completed a series of self-report scales measuring (1) perceived group entitativity, (2) intergroup emotion, and (3) engagement with the gender-polarized framing of the post. The survey concluded with demographic questions including age, gender, education level, marital status, and preferred social media platform.

The entire procedure was conducted online and took approximately 8 to 10 min to complete.

### 3.3 Materials and measures

#### 3.3.1 Stimulus materials

Participants viewed edited screenshots designed to closely reproduce the visual presentation of either RedNote or Zhihu. The stimuli incorporated platform-specific visual features, including layout, navigation elements, interaction icons, typography, and content presentation. Figure 1 provides a side-by-side comparison of the experimental stimuli and actual platform interfaces.

**Figure 1:**
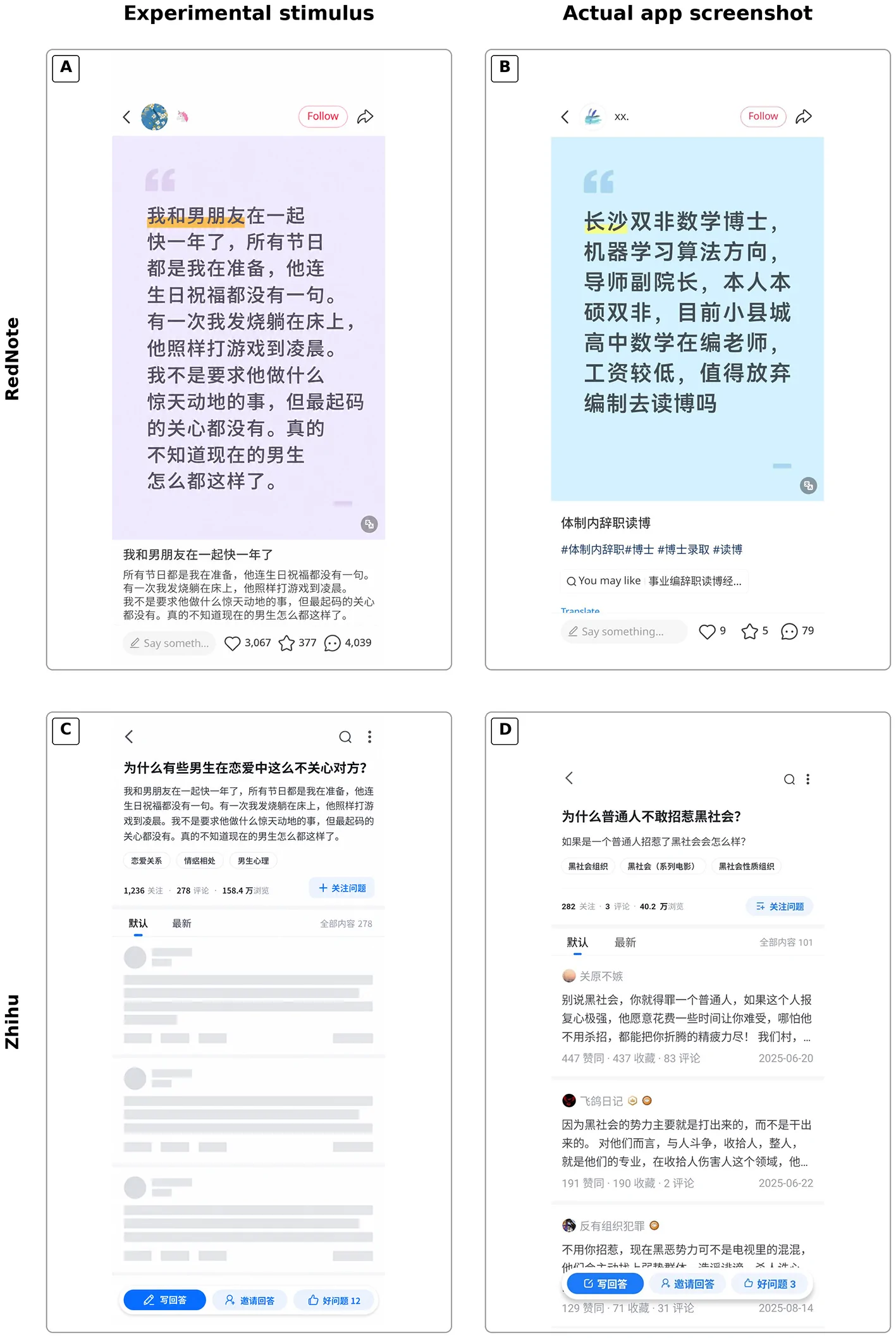
Comparison of experimental stimuli and actual platform interfaces. **(A)** Experimental stimulus presented in a RedNote-like interface. **(B)** Screenshot of the actual RedNote interface. **(C)** Experimental stimulus presented in a Zhihu-like interface. **(D)** Screenshot of the actual Zhihu interface.

To develop ecologically plausible stimulus materials, we first conducted an informal review of several hundred highly engaged gender-related posts on major Chinese social-media platforms, particularly RedNote and Zhihu. Posts were identified by browsing combinations of commonly used Chinese keywords related to gender and intimate relationships, including 男女关系 (male–female relationships), 恋爱 (romantic relationships), 男朋友/女朋友 (boyfriend/girlfriend), 两性话题 (gender/relationship topics), 情绪价值 (emotional support/value), 冷暴力 (silent treatment/emotional withdrawal), 家务 (housework), 家庭责任 (family responsibilities), 约会 (dating), and 恋爱开销 (dating expenses). Rather than reproducing individual posts verbatim, we used this review to identify recurring interpersonal-conflict themes and common ways in which personal experiences were generalized to men or women as a group.

Based on this review, we summarized the recurring content into four broad themes: emotional imbalance in romantic relationships, communication conflict, unequal household responsibilities, and negative evaluation following dating-related expenditures. We then constructed original vignettes representing each theme. Each vignette was designed to resemble the concise, first-person narrative style commonly observed in social-media posts while avoiding identifiable details or direct reproduction of existing content. Each participant viewed only one vignette theme, with the four themes equally distributed across the sample. The complete stimulus materials, including the original Chinese text and English translations for all gender-mirrored versions, are provided in Appendix A.

Each vignette was constructed in two gender-mirrored versions. In one version, a woman described a negative interaction with a male partner and generalized the incident to men; in the corresponding version, a man described the same type of interaction with a female partner and generalized the incident to women. The paired versions retained the same underlying interpersonal conflict and were designed to be broadly parallel in structure and meaning, while allowing gender-specific wording and contextual details to preserve natural and plausible scenarios. Importantly, the posts themselves contained an explicit gender-level generalization; therefore, the design assessed participants' responses to an already gender-generalizing frame rather than the spontaneous emergence of generalization from an exclusively individual-level incident.

This design was intended to maintain comparable interpersonal-conflict themes across the two gender-pairing conditions while preserving naturalistic gender-specific details. Target gender was matched to participant gender such that male participants viewed vignettes targeting women, whereas female participants viewed vignettes targeting men. Accordingly, differences between these conditions should be interpreted as gender-pairing differences rather than as independent effects of target gender, participant gender, or narrator gender.

Vignette theme was distinct from the experimental manipulations of target gender and platform. The four themes were distributed across the experimental conditions rather than being uniquely associated with particular target-gender or platform conditions. Thus, the four vignette themes should not be interpreted as four experimental conditions. The platform manipulation presented the assigned vignette in either the Zhihu or RedNote condition while retaining the substantive content of the vignette.

#### 3.3.2 Perceived group entitativity

Perceived entitativity was assessed using a 6-item scale adapted from Lickel et al. (2000). The items assessed perceived group unity, member similarity, common goals, interaction among members, importance of the group to its members, and temporal stability. Item wording was contextualized to refer specifically to the gender group targeted in the vignette. Example items included “This group is a real, unified group,” “Members of this group are similar to each other,” and “This group shares common goals.” Participants responded on a 7-point scale ranging from 1 (*strongly disagree*) to 7 (*strongly agree*). Responses were averaged to form a composite entitativity score, with higher scores indicating greater perceived entitativity. The scale demonstrated excellent internal consistency (Cronbach's α > 0.8).

#### 3.3.3 Intergroup emotion

Participants were asked to rate the degree to which they experienced eight different emotions (negative emotions: anger, disgust, contempt, fear, distrust; positive emotions: admiration, affection, trust) in response to the vignette, targeting the relevant gender group. Emotions were selected based on prior research on intergroup affect (Mackie et al., 2000; Paolini et al., 2004; Spears et al., 2002).

Each emotion was rated on a 7-point scale (1 = Not at all, 7 = Very strongly). Because the hypothesized affective pathway concerned negative emotional responses to gender-conflict content, the five negative emotions—anger, disgust, contempt, fear, and distrust—were averaged to form the negative intergroup-emotion composite used in the analyses. Higher scores therefore indicated stronger negative emotion toward the target gender group. The negative-emotion composite demonstrated good internal consistency (Cronbach's α > 0.80).

#### 3.3.4 Responses to gender-polarized framing

Drawing on the extended contact hypothesis (Wright et al., 1997), we developed five items to assess participants' responses to the gender-polarized framing presented in the posts. The items covered several related responses, including changes in overall group evaluation, perceived typicality of the focal individual, social distance, and reduced trust.

Example items included “This person's behavior changed how I think of the group,” “This person is typical of others in this group,” and “I feel less trusting toward this group after seeing this.” Participants responded on a 7-point scale ranging from 1 (strongly disagree) to 7 (strongly agree). Responses were averaged to form a composite score, with higher scores indicating stronger alignment with the negative, group-based framing presented in the post. The measure demonstrated excellent internal consistency (Cronbach's α > 0.90).

#### 3.3.5 Demographics and platform usage

Participants reported their gender (male, female, other), age, marital status, region (urban or rural), and highest level of education. Additionally, they were asked to indicate their typical usage pattern for RedNote and Zhihu (e.g., actively use one, both, or neither), which served as a check on ecological validity of platform assignment.

### 3.4 Analytical strategy

Analytical Strategy. All analyses were conducted in R version 4.5.0. First, composite scores were calculated for perceived entitativity, negative intergroup emotion, and responses to gender-polarized framing. Negative intergroup emotion was operationalized as the mean of anger, disgust, contempt, fear, and distrust ratings, and responses to gender-polarized framing was calculated as the mean of the five generalization response items.

To examine experimental-condition differences, separate 2 × 2 analyses of variance (ANOVAs) were conducted for perceived entitativity, negative intergroup emotion, and responses to gender-polarized framing, with gender and platform condition entered as between-subject factors. These models tested the main effects of target gender and platform condition as well as their interaction.

To examine the hypothesized indirect association between perceived entitativity and responses to gender-polarized framing through negative intergroup emotion, we estimated an observed-variable path model using the lavaan package. Perceived entitativity was specified as a predictor of negative intergroup emotion and responses to gender-polarized framing, and negative intergroup emotion was additionally specified as a predictor of responses to gender-polarized framing. Indirect, direct, and total effects were estimated using maximum likelihood estimation with 5,000 nonparametric bootstrap draws for standard errors.

The path model additionally adjusted for education, relationship status, and region. Education was coded as an ordinal variable ranging from 1 to 4. Relationship status was dummy coded with single participants as the reference category, and region was coded as 0 = prefecture-level city and 1 = county-level city or below.

All 800 completed responses were retained for analysis. The 800 participants were evenly distributed across the four experimental cells, with 200 participants in each gender-pairing × platform condition. The survey required responses to the study measures, and no cases were excluded for missing data or failed attention checks because no separate attention-check item was administered. Statistical significance was evaluated using two-sided tests with α = 0.05.

## 4 Results

### 4.1 Descriptive statistics

Participants reported relatively high perceived entitativity (M = 5.38, *SD* = 0.93), whereas mean levels of negative intergroup emotion (*M* = 3.58, *SD* = 0.85) and responses to gender-polarized framing (*M* = 3.53, *SD* = 0.36) were more moderate.

### 4.2 Effects of gender and platform on generalization

A 2 (Gender-pairing: male-target/female-participant vs. female-target/male-participant) × 2 (Platform: Zhihu vs. RedNote) between-subjects ANOVA was conducted to examine differences in responses to gender-polarized framing. A significant difference was observed between the two gender-pairing conditions, F _(1, 796)_ = 17.34, *p* < 0.001, η^2^ = 0.021. Responses to gender-polarized framing were stronger in the male-target/female-participant condition than in the female-target/male-participant condition.

Although statistically significant, this difference was small in magnitude (η^2^ = 0.021), accounting for approximately 2.1% of the variance in responses to gender-polarized framing. The main effect of platform was not significant, *F*_(1, 796)_ = 0.39, *p* = 0.533. The interaction between gender pairing and platform was also not significant, F _(1, 796)_ = 1.51, *p* = 0.220.

The estimated cell means were 3.59 [95% CI (3.54, 3.63)] for the male-target/female-participant RedNote condition, 3.45 [95% CI (3.40, 3.50)] for the female-target/male-participant RedNote condition, 3.57 [95% CI (3.52, 3.62)] for the male-target/female-participant Zhihu condition, and 3.50 [95% CI (3.45, 3.55)] for the female-target/male-participant Zhihu condition.

### 4.3 Path analysis with covariates

To examine the associations among perceived entitativity, negative intergroup emotion, and responses to gender-polarized framing, we estimated an observed-variable path model. The model tested whether the association between perceived entitativity and attitude generalization included a statistically detectable indirect association through negative intergroup emotion. In this model, negative intergroup emotion was specified as a potential indirect pathway between perceived entitativity and generalization, with education level, marital status, and geographic region included as covariates. Marital status was dummy coded, with “single” as the reference category, and region was recoded into a binary variable distinguishing county-level areas (1) from prefecture-level cities (0). The model was estimated using maximum likelihood with 5,000 bootstrapped samples.

Figure 2 illustrates the observed-variable path model with standardized coefficients.

**Figure 2:**
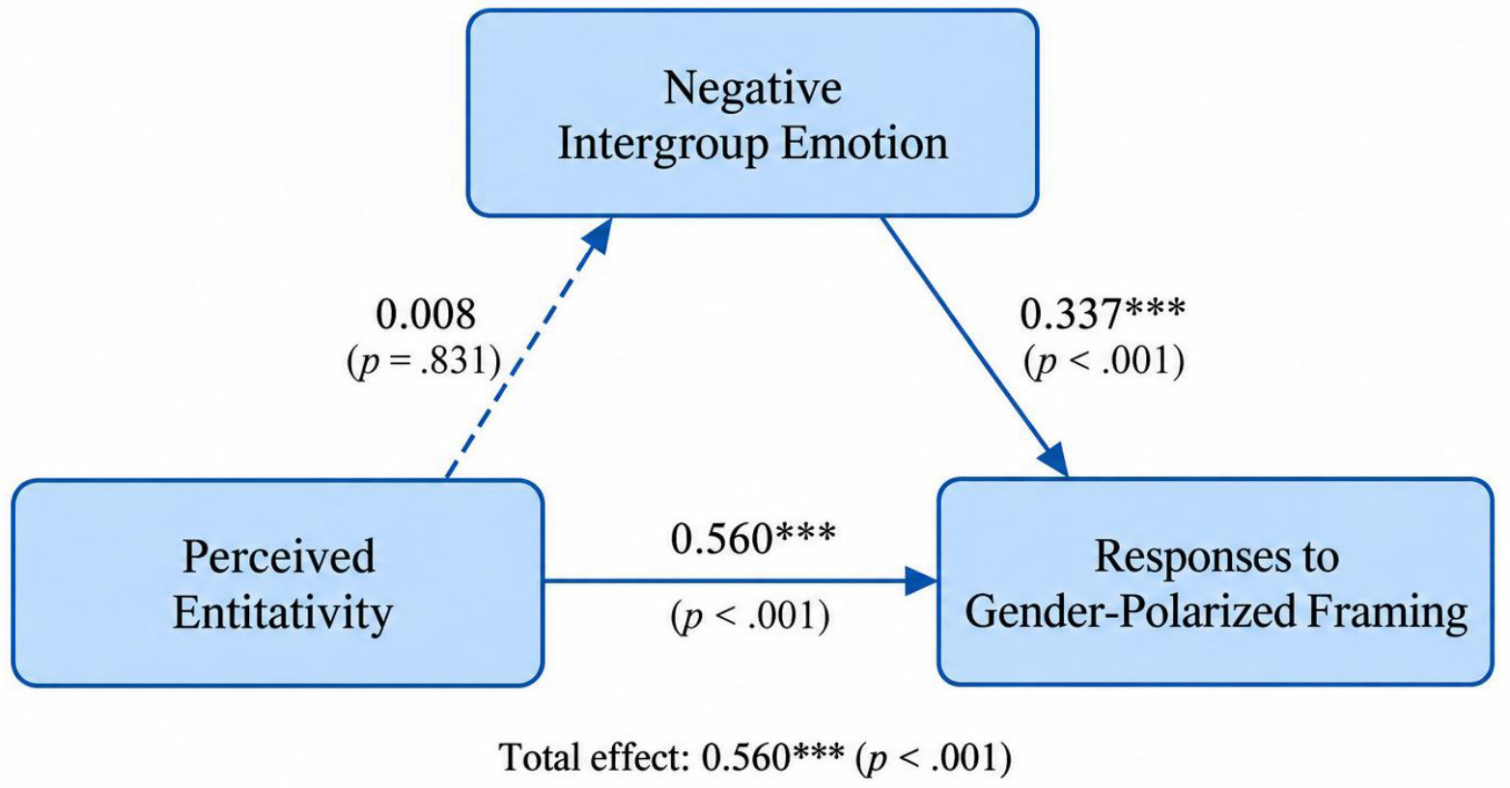
Observed-variable path model of entitativity, negative intergroup emotion, and attitude generalization. Note: Standardized coefficients are shown. (education, relationship status, and region) are included in the model but are not displayed for simplicity. ^***^p < 0.001.

Perceived entitativity was positively associated with responses to gender-polarized framing, whereas the estimated indirect association through negative intergroup emotion was not significant. The association between perceived entitativity and attitude generalization was significant and positive, [*B* = 0.256, *SE* = 0.012, β = 0.560, *p* < 0.001, 95% CI (0.232, 0.280)], which showed stronger alignment with the negative group-based framing presented in the post. The association between perceived entitativity and negative intergroup emotion was not significant [*B* = 0.004, SE = 0.017, β = 0.008, *p* = 0.829, 95% CI (−0.029,0.037)]. Meanwhile, negative intergroup emotion was positively associated with responses to gender-polarized framing [*B* = 0.339, SE = 0.028, β = 0.337, *p* < 0.001, 95% CI (0.284, 0.394)]. However, the estimated indirect association through negative intergroup emotion was not statistically significant [*B* = 0.001, SE = 0.006, β =0.003, *p* = 0.830, 95% CI (−0.011, 0.013)]. Thus, the data did not provide evidence that the association between entitativity and generalization operated indirectly through negative emotion.

Among the control variables, most were not significantly associated with the endogenous variables, except that being divorced was associated with lower negative intergroup emotion (β = −0.097, *p* = 0.005). The model accounted for 43.1% of the variance in responses to gender-polarized framing (*R*^2^ =0.431) and 1.2% of the variance in negative intergroup emotion (*R*^2^ =0.012).

## 5 Discussion

The present study examined why exposure to an individual gender-conflict incident can translate into broader judgments of men or women as a group. Perceived entitativity emerged as the strongest correlate of responses to gender-polarized framing: individuals who perceived the target gender as a more coherent and unified social entity showed stronger alignment with the negative group-based framing presented in the posts. Negative intergroup emotion was also positively associated with responses to gender-polarized framing, but the estimated indirect association through emotion was not significant. In addition, responses differed modestly across the gender-pairing conditions, whereas neither the main effect of platform context nor the gender-pairing × platform interaction was significant. Together, these findings suggest that responses to gender-polarized discourse are associated with both cognitive perceptions of groupness and negative affect, while providing no support for the hypothesized sequential pathway from entitativity through negative emotion.

The relationship between entitativity and responses to gender-polarized framing extends existing theories of group perception in an important direction. Entitativity theory proposes that groups perceived as coherent entities are more likely to elicit group-level rather than person-level inference (Campbell, 1958; Lickel et al., 2000). Recent research similarly suggests that entitativity facilitates the generalization of information about individual group members to the broader group and can contribute to collective blame following individual misconduct (Alam et al., 2025; Neji et al., 2025). The present findings extend this perspective to responses to explicitly gender-generalizing discourse in online communication. Unlike small interactive groups or organizations, men and women are extremely large and internally heterogeneous social categories whose members do not share common goals or coordinated behavior. Nevertheless, variation in perceived gender entitativity was strongly associated with the extent to which participants aligned with posts that framed an interpersonal conflict in group-based gender terms. Thus, the psychological relevance of entitativity may extend beyond objectively cohesive groups: even for broad and heterogeneous categories such as gender, perceiving greater group coherence may make explicitly group-based interpretations of interpersonal conflict more compelling.

A second contribution concerns the relationship between cognitive group perception and intergroup emotion. Negative intergroup emotion was independently associated with stronger responses to gender-polarized framing, consistent with research emphasizing the role of affect in intergroup judgment and online polarization. However, entitativity did not significantly predict emotional responses, and the indirect effect through emotion was negligible. The results therefore do not support the hypothesized sequential pathway in which perceiving a group as more unified generates stronger negative emotion, which subsequently strengthens alignment with gender-polarized framing. Instead, the findings are more consistent with partially distinct associations of cognitive group perception and negative affect with responses to polarized gender discourse. Negative emotion may increase the salience or evaluative force of a gender-conflict post, whereas perceived entitativity may make its group-based framing appear more plausible or representative. This distinction is theoretically important because it suggests that reducing emotional hostility alone may not eliminate alignment with polarized group-based interpretations when perceptions of gender-group coherence remain high.

These findings also contribute to the broader literature on gender backlash and gender polarization. Research on contemporary gender conflict has documented increasingly adversarial gender narratives across both online and offline settings. The present results complement this literature by highlighting an audience-level psychological process that may help sustain such discourse. Polarized gender narratives do not depend only on those who produce or disseminate antagonistic content; their persistence may also depend on whether audiences perceive broad gender categories as sufficiently coherent for an individual member's behavior to appear informative about the group as a whole. From this perspective, perceived gender entitativity may help connect everyday interpersonal conflict with broader gender-group antagonism.

At the same time, the present findings should not be interpreted as suggesting that gender polarization takes the same form across societies. The specific interpersonal conflicts represented in our stimuli—including emotional investment, communication, household responsibilities, and dating-related expenditures—were derived from Chinese social-media discourse and are embedded in the contemporary Chinese cultural context. Comparable gender conflicts in South Korea, Japan, Europe, or the United States may center on different issues and may be shaped by different social norms, gender-role expectations, and digital cultures. What may be more transferable across contexts is the social-cognitive process examined here: when an interpersonal incident is already framed as evidence about a broader gender category, individuals who perceive that category as more coherent may be more receptive to such generalization. The present findings therefore provide a basis for comparative rather than universal claims. Future cross-cultural research using locally grounded gender-conflict stimuli could examine whether the association between perceived entitativity and responses to gender-polarized framing generalizes across East Asian, Western, and other sociocultural contexts.

The experimental-condition results provide limited evidence for platform differences but indicate a modest difference between the two gender-pairing conditions. Platform context showed neither a significant main effect nor a significant interaction with gender pairing. Thus, presenting the same broad type of gender-conflict content within RedNote-like vs. Zhihu-like visual contexts did not produce detectable differences in participants' responses in the present study. Responses were somewhat stronger in the male-target/female-participant condition than in the female-target/male-participant condition. However, because participant gender, narrator gender, and target gender were structurally paired in the design, this difference cannot be attributed uniquely to any one of these factors.

The absence of a platform effect does not imply that platform characteristics are irrelevant to gender-polarized discourse more generally. Prior research suggests that gender antagonism may take different forms across Chinese social-media platforms as a function of platform affordances, community norms, and patterns of interaction (Su et al., 2026). The present manipulation, however, isolated platform-specific visual presentation while holding the substantive content of the posts broadly constant. It therefore provides a relatively narrow test of platform context rather than a test of the full social and algorithmic environments of RedNote and Zhihu. Accordingly, explanations based on emotional expressiveness, community composition, algorithmic exposure, or platform culture should be regarded as possibilities for future investigation rather than established mechanisms in the present study.

The present study therefore makes three principal theoretical contributions. First, it extends entitativity-based accounts of group perception to explicitly gender-polarized online discourse. The findings suggest that even for broad and heterogeneous social categories such as gender, perceiving greater group coherence is associated with stronger alignment with group-based interpretations of interpersonal conflict. Second, the study distinguishes perceived groupness from negative affect as related but non-sequential correlates of responses to gender-polarized framing. Although both entitativity and negative intergroup emotion were associated with stronger responses, the hypothesized indirect pathway through emotion was not supported. Third, the study shifts attention from the emergence of gender generalization to individuals' responses to discourse in which such generalization is already embedded. This distinction is particularly relevant to social-media environments, where users frequently encounter interpersonal experiences that are already framed as evidence about men or women as broader social categories.

These findings may also have implications for efforts to reduce gender polarization online. Interventions could consider addressing not only emotional hostility but also perceptions that broad social categories such as gender are highly homogeneous or internally coherent. Digital-literacy approaches that encourage users to distinguish individual experiences from group-level evidence may be particularly relevant when social-media posts explicitly invite such generalization. At the same time, the outcome examined in the present study reflects psychological responses to gender-polarized framing rather than actual online participation. The findings therefore should not be interpreted as showing that greater entitativity or negative emotion necessarily causes users to comment, share, attack others, or otherwise participate behaviorally in online gender conflict. Future research incorporating behavioral outcomes could examine whether the psychological alignment observed here translates into active reinforcement or dissemination of polarized discourse.

Several limitations should be considered when interpreting these findings. First, the experimental posts themselves contained explicit gender-level generalizations. Accordingly, the study cannot determine whether participants spontaneously generalized from an individual incident to the broader gender group; rather, it examines variation in responses to an already gender-generalizing frame. Second, participant gender, narrator gender, and target gender were structurally paired, such that female participants viewed female-narrated posts targeting men and male participants viewed male-narrated posts targeting women. The observed gender-pairing difference therefore cannot be uniquely attributed to participant gender, narrator gender, or target gender. Third, although the gender-mirrored vignettes were designed around parallel interpersonal-conflict themes, some gender-specific contextual details differed. Their psychological equivalence in perceived severity, plausibility, or social meaning was not independently validated, and these differences may have contributed to the observed gender-pairing effect. Fourth, although the experimental screenshots were designed to reproduce recognizable visual features of RedNote and Zhihu, no formal manipulation check was administered to determine whether participants recognized or psychologically differentiated the intended platform contexts. The null platform findings should therefore be interpreted with this limitation in mind.

Measurement limitations also warrant consideration. The response-to-framing composite included several related but conceptually distinguishable responses, including changes in group evaluation, perceived typicality, social distance, and reduced trust. Although these items showed high internal consistency, the present study did not independently establish their dimensional structure or broader construct validity. The composite should therefore be interpreted as a broad index of responses to gender-polarized framing rather than as a validated measure of a single underlying construct. In addition, distrust was included in the negative intergroup-emotion composite, whereas reduced trust was also represented in the response-to-framing measure, creating some conceptual overlap between the two measures. The positive association between negative intergroup emotion and responses to gender-polarized framing should consequently be interpreted with appropriate caution. Finally, entitativity, emotion, and responses to framing were measured after exposure to the posts. Although the path model was theoretically specified in a directional manner, the observed associations do not establish the temporal or causal ordering among these psychological responses.

Future research could address these limitations by independently manipulating or balancing participant, narrator, and target gender; pretesting gender-mirrored scenarios for perceived severity and psychological equivalence; incorporating explicit platform-recognition checks; and developing more differentiated measures of cognitive, affective, and behavioral responses to gender-polarized discourse. It would also be valuable to examine actual behavioral responses, such as liking, commenting, sharing, or producing similarly generalized content. Such designs could more directly test how psychological alignment with gender-polarized framing develops into active participation in online gender conflict.

## 6 Conclusion

The present study examined how perceived group entitativity and negative intergroup emotion are associated with individuals' responses to gender-polarized social-media posts. Participants who perceived the target gender as a more coherent and unified social entity showed stronger alignment with the negative group-based framing presented in the posts. Negative intergroup emotion was also positively associated with these responses, although the hypothesized indirect pathway through emotion was not supported. Platform context showed no significant effect, while the modest difference between gender-pairing conditions cannot be attributed uniquely to participant gender, narrator gender, or target gender because these factors were structurally paired.

More broadly, the findings shift attention from how gender generalization initially emerges to how individuals respond when such generalization is already embedded in online discourse. Social-media posts often frame particular interpersonal experiences as evidence about men or women more broadly, and the present findings suggest that perceived group coherence may be particularly relevant to how strongly individuals align with such framing. Understanding why some users align more strongly with already-polarized gender narratives may therefore provide one piece of the broader explanation for how “gender wars” are psychologically sustained in online environments.

## Data Availability

The raw data supporting the conclusions of this article will be made available by the authors, without undue reservation.
