# Supplementary material for: Joining the gender war online: entitativity, negative emotion, and engagement with gender-polarized posts
Source: Front Psychol. 2026 Oct 26;17:1961949. doi: 10.3389/fpsyg.2026.1961949 (PMC13745684; doi:10.3389/fpsyg.2026.1961949)
Supplement: Supplementary file 1 [file Data_Sheet_1.docx]

附录 A：实验问卷（中英文对照）

引导语 / Introduction

本研究旨在了解人们对网络平台中性别相关内容的感知与态度。以下是一段来自社交平台的改编内容，请您阅读后完成问卷。
This study investigates how people perceive and respond to gender-related content on social media platforms. Please read the following adapted post and complete the questionnaire.

实验材料 / Stimulus Material

场景 1：情感付出失衡 Emotional Imbalance in Relationships

女批男版 / Female criticizes male

我和男朋友在一起快一年了，所有节日都是我在准备，他连生日祝福都没有一句。有一次我发烧躺在床上，他照样打游戏到凌晨。我不是要求他做什么惊天动地的事，但最起码的关心都没有。真的不知道现在的男生怎么都这样了。

My boyfriend and I have been together for almost a year, but I’m the one who prepares for every holiday, and he hasn’t even said “happy birthday” to me. Once, I had a fever and was lying in bed, but he still played video games until late at night. I’m not asking him to do anything earth‑shattering, but at least a little care would be nice. I really don’t understand why guys are like this nowadays.

男批女版 / Male criticizes female

我和女朋友在一起快一年了，所有节日都是我在准备，她连生日祝福都没有一句。有一次我发烧躺在床上，她照样刷手机刷到凌晨。我不是要求她做什么惊天动地的事，但最起码的关心都没有。真的不知道现在的女生怎么都这样了。

My girlfriend and I have been together for almost a year, but I’m the one who prepares for every holiday, and she hasn’t even said “happy birthday” to me. Once, I had a fever and was lying in bed, but she still scrolled through her phone until late at night. I’m not asking her to do anything earth‑shattering, but at least a little care would be nice. I really don’t understand why girls are like this nowadays.

场景 2：沟通冲突 Communication Conflict

女批男版 / Female criticizes male

每次我和男朋友沟通问题，他不是沉默就是冷暴力。我好好说话，他却觉得我在“情绪化”。我说的不对你可以讲，但别老装作听不见。现在的男生是不是真的都不懂怎么沟通？

Every time I try to communicate with my boyfriend, he either clams up or gives me the silent treatment. I speak calmly, but he thinks I’m being “emotional.” If I say something wrong, you can tell me, but don’t keep pretending you can’t hear me. Do guys nowadays really not know how to communicate at all?

男批女版 / Male criticizes female

每次我和女朋友沟通问题，她不是沉默就是冷暴力。我好好说话，她却觉得我在“情绪化”。我说的不对你可以讲，但别老装作听不见。现在的女生是不是真的都不懂怎么沟通？

Every time I try to communicate with my girlfriend, she either clams up or gives me the silent treatment. I speak calmly, but she thinks I’m being “emotional.” If I say something wrong, you can tell me, but don’t keep pretending you can’t hear me. Do girls nowadays really not know how to communicate at all?

场景 4：家庭责任不平衡 Household Responsibility Imbalance

女批男版 / Female criticizes male

结婚以后几乎什么都我一个人来，工作、做饭、照顾孩子。他一下班就躺着刷手机，说他两句还不耐烦。我不是你妈，也不是你佣人。现在的男生到底有没有一点责任感？

After we got married, I’m basically doing everything by myself – working, cooking, taking care of the kids. The moment he gets off work, he lies down and scrolls through his phone, and if I say a word to him, he gets annoyed. I’m not your mother, nor your maid. Do guys nowadays have any sense of responsibility at all?

男批女版 / Male criticizes female

结婚以后几乎什么都我一个人来，工作、做饭、照顾孩子。她一下班就躺着刷手机，说她两句还不耐烦。我不是你爸，也不是你佣人。现在的女生到底有没有一点责任感？

After we got married, I’m basically doing everything by myself – working, cooking, taking care of the kids. The moment she gets off work, she lies down and scrolls through her phone, and if I say a word to her, she gets annoyed. I’m not your father, nor your maid. Do girls nowadays have any sense of responsibility at all?

场景 5：朋友圈开销与被评价 Dating Expenses and Being Judged

女批男版 / Female criticizes male

跟男朋友出去吃饭，我自掏腰包请客。他事后跟朋友说“她太主动了，有点黏人”。我真的无语，花钱请你吃饭不行？怎么现在的男生动不动就把女生标签化？

When I went out to eat with my boyfriend, I paid for the meal out of my own pocket. Afterwards, he told his friends, “She’s too forward, a bit clingy.” I was speechless. So it’s wrong to treat you to a meal? Why do guys nowadays label girls so easily?

男批女版 / Male criticizes female

跟女朋友出去吃饭，我自掏腰包请客。她事后跟朋友说“他太主动了，有点黏人”。我真的无语，花钱请你吃饭不行？怎么现在的女生动不动就把男生标签化？

When I went out to eat with my girlfriend, I paid for the meal out of my own pocket. Afterwards, she told her friends, “He’s too forward, a bit clingy.” I was speechless. So it’s wrong to treat you to a meal? Why do girls nowadays label guys so easily?

部分 I：实体性感知 / Perceived Entitativity

1. 这个群体是一个真实的、统一的群体。
This group is a real, unified group.
(1 = 完全不同意 / Strongly Disagree, 7 = 非常同意 / Strongly Agree)

2. 这个群体成员彼此之间很相似。
Members of this group are similar to each other.
(1 = 完全不同意 / Strongly Disagree, 7 = 非常同意 / Strongly Agree)

3. 这个群体有明确的共同目标。
This group shares common goals.
(1 = 完全不同意 / Strongly Disagree, 7 = 非常同意 / Strongly Agree)

4. 这个群体的成员经常互动。
Members of this group interact frequently.
(1 = 完全不同意 / Strongly Disagree, 7 = 非常同意 / Strongly Agree)

5. 这个群体对其成员很重要。
This group is important to its members.
(1 = 完全不同意 / Strongly Disagree, 7 = 非常同意 / Strongly Agree)

6. 这个群体会长期存在。
This group stays together over time.
(1 = 完全不同意 / Strongly Disagree, 7 = 非常同意 / Strongly Agree)

部分 II：群体情绪 / Intergroup Emotions

请评价你此刻对该性别群体（男性/女性）的情绪感受：
Please rate how strongly you feel each of the following emotions toward the gender group.

- 愤怒 Anger (1 = 完全没有 / Not at all, 7 = 非常强烈 / Very strongly)

- 厌恶 Disgust (1 = 完全没有 / Not at all, 7 = 非常强烈 / Very strongly)

- 蔑视 Contempt (1 = 完全没有 / Not at all, 7 = 非常强烈 / Very strongly)

- 害怕 Fear (1 = 完全没有 / Not at all, 7 = 非常强烈 / Very strongly)

- 不信任 Distrust (1 = 完全没有 / Not at all, 7 = 非常强烈 / Very strongly)

- 喜爱 Affection (1 = 完全没有 / Not at all, 7 = 非常强烈 / Very strongly)

- 钦佩 Admiration (1 = 完全没有 / Not at all, 7 = 非常强烈 / Very strongly)

- 信任 Trust (1 = 完全没有 / Not at all, 7 = 非常强烈 / Very strongly)

部分 III：态度泛化 / Attitude Generalization

1. 这个人的行为改变了我对该群体的看法。
This person’s behavior changed how I think of the group.
(1 = 完全不同意 / Strongly Disagree, 7 = 非常同意 / Strongly Agree)

2. 这段内容让我对该群体整体印象更负面了。
This post made me feel more negative about this group.
(1 = 完全不同意 / Strongly Disagree, 7 = 非常同意 / Strongly Agree)

3. 我认为这个人是该群体的典型代表。
This person is typical of others in this group.
(1 = 完全不同意 / Strongly Disagree, 7 = 非常同意 / Strongly Agree)

4. 我更倾向于对这个群体保持距离。
I would prefer to distance myself from this group now.
(1 = 完全不同意 / Strongly Disagree, 7 = 非常同意 / Strongly Agree)

5. 看到这段内容后我更不信任该群体。
I feel less trusting toward this group after seeing this.
(1 = 完全不同意 / Strongly Disagree, 7 = 非常同意 / Strongly Agree)

部分 IV：背景信息 / Demographics

你的性别是？
What is your gender?
- 男 Male
- 女 Female
- 其他 Other

你的年龄是？
What is your age? （请填写具体数字 Please enter a number）

你最常使用的社交平台情况是(日均使用半小时以上为活跃)？
Which best describes your current platform use?
- 主要使用小红书 Active on Xiaohongshu
- 主要使用知乎 Active on Zhihu
- 两者都活跃 Active on both
- 两者都不常用 Inactive on both

你的最高学历是？
What is your highest level of education?
- 高中及以下 High school or below
- 大专 Vocational college
- 本科 Bachelor’s degree
- 研究生及以上 Master’s or above

你目前的婚恋状态是？
What is your current relationship status?
- 单身 Single
- 恋爱中 In a relationship
- 已婚 Married
- 离异或其他 Divorced or other

你目前的常住地区属于：
Your current residence is in:
- 地级市 Prefecture-level city
- 县级市或以下 County-level or below
